# Modulating Drug Resistance by Targeting *BCRP/ABCG2* Using Retrovirus-Mediated RNA Interference

**DOI:** 10.1371/journal.pone.0103463

**Published:** 2014-07-30

**Authors:** Ni Xie, Lisha Mou, Jianhui Yuan, Wenlan Liu, Tingting Deng, Zigang Li, Yi Jin, Zhangli Hu

**Affiliations:** 1 Shenzhen Second People’s Hospital, The First Affiliated Hospital of Shenzhen University, Shenzhen, Guangdong, China; 2 The Shenzhen Center for Disease Control and Prevention, Shenzhen, Guangdong, China; 3 The Shenzhen Graduate School, Peking University, Shenzhen, Guangdong, China; 4 College of Life Science, Shenzhen University, Shenzhen, Guangdong, China; University of Navarra, Spain

## Abstract

**Background:**

The BCRP/ABCG2 transporter, which mediates drug resistance in many types of cells, depends on energy provided by ATP hydrolysis. Here, a retrovirus encoding a shRNA targeting the ATP-binding domain of this protein was used to screen for highly efficient agents that could reverse drug resistance and improve cell sensitivity to drugs, thus laying the foundation for further studies and applications.

**Methodology/Principal Findings:**

To target the ATP-binding domain of *BCRP/ABCG2*, pLenti6/BCRPsi shRNA recombinant retroviruses, with 20 bp target sequences starting from the 270^th^, 745^th^ and 939^th^ bps of the 6^th^ exon, were constructed and packaged. The pLenti6/BCRPsi retroviruses (V-BCRPi) that conferred significant knockdown effects were screened using a drug-sensitivity experiment and flow cytometry. The human choriocarcinoma cell line JAR, which highly expresses endogenous *BCRP/ABCG2*, was injected under the dorsal skin of a hairless mouse to initiate a JAR cytoma. After injecting V-BCRPi-infected JAR tumor cells into the dorsal skin of hairless mice, *BCRP/ABCG2* expression in the tumor tissue was determined using immunohistochemistry, fluorescent quantitative RT-PCR and Western blot analyses. After intraperitoneal injection of *BCRP/ABCG2-*tolerant 5-FU, the tumor volume, weight change, and apoptosis rate of the tumor tissue were determined using in situ hybridization. V-BCRPi increased the sensitivity of the tumor histiocytes to 5-FU and improved the cell apoptosis-promoting effects of 5-FU in the tumor.

**Conclusions/Significance:**

The goal of the *in vivo* and *in vitro* studies was to screen for an RNA interference recombinant retrovirus capable of stably targeting the ATP-binding domain of *BCRP/ABCG2* (V-BCRPi) to inhibit its function. A new method to improve the chemo-sensitivity of breast cancer and other tumor cells was discovered, and this method could be used for gene therapy and functional studies of malignant tumors.

## Introduction

The ultimate goals of oncology drug resistance mechanism research are to find targets of drug resistance and screen for specific agents that can reverse these phenotypes, to improve the curative effects of chemotherapy for prevention and clinical treatment, and to reduce drug toxicity. Several papers have demonstrated the reversal of BCRP/ABCG2 multidrug resistance as well as specific, non-specific and chemical drug resistance [1]. The first BCRP/ABCG2 inhibitor was fumitremorgin C (FTC) [2]. The same reversal effects have been observed after targeting other membrane transporter proteins due to the structural similarities of these proteins [3]. For example, one inhibitor, isoquinoline acridorex (GF120918), and an analogue of dipyridamole, BIB-E, helped to inhibit the drug resistance phenotype mediated by BCRP/ABCG2 [4], [5]. Typically reversal-agent studies are performed for specific targets and unitary target structures. However, these studies have unclear clinical application prospects and weak feasibility. Studying drug-resistance mechanisms and reversal agents with the same inhibitory effects is both valuable and important for understanding the multidrug-resistance phenotype.

A previous study showed that ATP participates in multiple processes and is hydrolyzed to provide direct or indirect energy for membrane proteins to transport their substrates [6]. Thus, to inhibit the BCRP/ABCG2 transporter, a prospective drug should target ATP hydrolysis [7], [8]. P-glycoprotein (P-gp) and other classical membrane proteins that belong to the ATP-binding cassette (ABC) membrane transporter protein family [9]–[13] require energy provided by ATP hydrolysis. In this study, a shRNA-encoding retrovirus with high infection efficiency was used to stably express a hairpin structure targeting the ABC domain of *BCRP/ABCG2*, and cell and animal experiments were conducted. The goal of the study was to screen for constructs that could efficiently target the ATP-binding domain and for agents that could reverse the drug-resistant phenotypes, which would ideally improve tumor cell sensitivity to drugs and lay a solid foundation for further studies and applications.

## Materials and Methods

### Cells, strains, plasmids and reagents

The 293FT virus-packaging cells, *Escherichia coli* TOP10 cells, Stbl3 competent cells, pENTR/U6, and the shRNA retrovirus vector pLenti6/BLOCK-iT-DEST were purchased from Invitrogen (Carlsbad, CA, USA). The human choriocarcinoma cell line JAR was purchased from the Shanghai Institute of Cellular Biology of the Chinese Academy of Sciences. BALB/c-nu/nu hairless mice (3- to 5-week-old females) were provided by the Laboratory Animal Center at the Guangzhou University of Traditional Chinese Medicine. The mice were raised in a specific pathogen-free environment. All animal experiments were conducted in accordance with the NIH Guide for the Care and Use of Laboratory Animals, and the methods were approved by the Ethics Committee at the Shenzhen Center for Disease Control and Prevention. The DNA loading buffer, DNA molecular weight marker, and PCR kit were purchased from Takara (TaKaRa, Dalian, China), and the agarose was obtained from BBI (Toronto, Ontario, Canada). RPMI-1640 medium, DMEM, penicillin/streptomycin double-resistant fluid, 0.25% trypsin-EDTA and fetal calf serum were obtained from Gibco (Grand Island, NY, USA). The single-stranded oligonucleotides and primers were synthesized by Takara. The HRP-conjugated goat anti-mouse secondary antibody was obtained from Sigma (Shanghai, China). The tris (hydroxymethyl) aminomethane (Tris), acrylamide, dithiothreitol (DTT) and tetramethylethylenediamine (TEMED) were purchased from Sangon Biotech Co., Ltd (Shanghai, China). The Western blotting prestain was obtained from Fermentas (Hanover, MD, USA). The rabbit anti-*β*-actin primary antibody was obtained from Biovision ((Mountain View, CA, USA), and the mouse anti-BCRP primary antibody (BXP-21) was purchased from Alexis (San Diego, CA, USA). The ECL and 2-D quantification kits were obtained from GE (Uppsala. Sweden). Trizol reagent was obtained from Invitrogen, and the reverse transcription and fluorogenic quantitative PCR kits were purchased from Takara. The DAPI was obtained from APPLICHEM (Darmstadt, Germany). The paraformaldehyde was domestic and analytically pure, and the 5-fluorouracil (5-FU) was domestic and obtained from SunnyHope Pharmaceutical Co., Ltd (Chengdu, China). The Cellular Orthotopic apoptosis detection kit was obtained from Shanghai KeyGEN Biotech Co., Ltd (Shanghai, China). All other chemical reagents were domestic, analytical reagents.

### Design and synthesis of *BCRP/ABCG2* RNA interference constructs

The BLOCK-iT RNAi Designer at Invitrogen was used to design DNA oligonucleotides complementary to the ABC region of the *BCRP/ABCG2* gene, beginning at the 207^th^, 745^th^, and 939^th^ initiation sites of the 6^th^ exon. These oligonucleotides were then synthesized, and the sequences of each pair of DNA oligonucleotides included the following sequences: upstream sequence - target sense sequence of 5′-CACCG-20 bases and antisense sequence of 3′-CGAA-20 bases; downstream sequence - antisense sequence of -C-3′ and 5′-AAAA-44, which is complementary to the upstream sequence.

### Construction of the pENTR/U6-BCRPi entry vector

At room temperature, 5 µl of “Top strand” DNA oligo (200 µM), 5 µl of “Bottom strand” DNA oligo (200 µM), 2 µl of 10× Oligo Annealing Buffer, and 8 µl of DNase/RNAse-free water (for a total volume of 20 µl) was placed in a 0.5-ml sterile EP centrifuge tube. The reaction mixture was incubated at 95°C for 4 minutes. In total, 99 µl of DNase/RNAse-free water was added to 1 µl of each of the double-strand products (500 nM) to dilute them 100-fold, resulting in a final concentration of 5 nM. The samples were then mixed with 4 µl of 5× Ligation Buffer, 2 µl of pENTR/U6 (0.5 ng/µl), 1 µl of double-stranded oligo (5 nM), 12 µl of DNase/RNAse-free water, and 1 µl of T4 DNA Ligase (1 U/µl), which resulted in 20 µl of reactive liquid that was then incubated for two hours at room temperature. Competent *E. coli* cells were thawed and added to the recombinant plasmid; this mixture was then incubated on ice for 10 minutes, heat shocked for 30 seconds at 42°C, and then immediately placed on ice. In total, 250 µl of room-temperature S.O.C. medium was then added to the cells, and the culture was shaken slightly for one hour at 37°C. Ampicillin was added to a sterile LA plate to serve as the negative control, and kanamycin was added to the other plates. Each *E. coli* transformation mixture consisted of a volume of 250 µl, and 125 µl was used to coat the plate. The plates were then incubated for 20 minutes in a 37°C incubator, and the plate was then turned over and incubated for an additional 16 hours. Positive clones were selected for sequencing.

### Construction of the pLenti6/BCRPi RNAi expression vector and preparation of recombinant retrovirus

The pLenti6/DEST vector (6 µg) was dissolved in 40 µl of TE buffer (pH 8.0) at a final concentration of 150 ng/µl. A 30-µl recombination mixture was established, and 2 µl of *LR Clonase II* enzyme was added. The mixture was then incubated for 18 hours at 25°C. Proteinase K (1 µl) was added, and the mixture was incubated for 1 hour; 5 µl of the reaction product was mixed with competent *E. coli* cells. The bacterial mixtures were then incubated on ice for 30 minutes, heat shocked for 45 seconds at 42°C, and placed on ice for 2 minutes. In total, 250 µl of S.O.C. medium was then added to the cells, and the culture was incubated for one hour at 37°C. The transformation reactions were plated, and the bacterial colonies were observed after incubation at 37°C. Positive clones were then selected for sequencing. 293FT cells were cultured at 37°C in 5% CO_2_ until they reached 90% confluency. Next, 195 µg of Virapower was placed in 195 µl of sterile TE buffer, and 9 µg of Virapower Packing Mix was mixed with 3 µg of pLenti6/BLOCK-iT-DEST Lentiviral RNAi. Lipofectamine 2000 was added, and the solution was incubated for 20 minutes at room temperature. The 293FT cells were incubated overnight at 37°C in 5% CO_2_. After cotransfection for 48 to 72 hours, the cells were centrifuged at 1000 rpm for 10 minutes, and the supernatant, which contained the virus, was collected. The retrovirus supernatant was then added to the cells once they reached 30–50% confluency. After overnight culturing, the cell culture medium was removed, and the cells were washed with PBS. Methylrosanilinium chloride solution (1 ml) was added, and the cells were incubated for 10 minutes at room temperature. The number of blue clones was then calculated, and the titers of the retroviruses were obtained by multiplying the average value by the dilution factor (unit: TU/ml).

### Cell culture

The culture solution was RPMI-1640 medium supplemented with 10% fetal calf serum and a pH value of 7.2 to 7.4. The cells were cultured in a 37°C incubator with 5% CO_2_. Once the cells adhered to the plate, the medium was exchanged every 2 to 3 days. When the cells reached 80–90% confluency, they were washed once with PBS, Next, 1 ml of 0.25% pancreatin was added slowly to the plates until the entire cell surface was covered, and the cells were incubated with the digestive fluid at room temperature for 5 to 10 minutes. Cytoplasm retraction was observed under a microscope, and new solution was added until the cell space enlarged. The cells were manually detached, inoculated into 2 to 3 culture flasks and moved to a 37°C incubator with 5% CO_2_ to allow for growth.

### Drug sensitivity after V-BCRPi infection, measured by cell survival

After JAR cells infected with each recombinant V-BCRPi retrovirus were incubated in 96-well cell culture plates for 48 hours, the IC_50_ concentration of 5-FU (60 mg/l) was added to the culture, and the cells were incubated for 72 hours. A blank cell group and cells incubated with the BCRP/ABCG2-specific inhibitor Ko143 (final concentration: 10 µmol/l) were used as experimental control groups. The survival ratios of the cells were then determined.

### *BCRP/ABCG2* expression in JAR cells measured by fluorescent quantitative RT-PCR and Western blot analyses

After infecting JAR cells with each recombinant V-BCRPi vector for 48 hours, RNA was extracted from the cells to determine the gene expression levels using fluorescent quantitative RT-PCR. Blank cell groups were used as controls in these experiments. In addition, whole cell extracts were collected. After the quantitative measurements, SDS-PAGE protein electrophoresis, transfer, primary and secondary antibody incubation, ECL chemiluminescence detection and photographic fixing, the protein expression levels were determined. The blank cell groups were used as controls in these experiments.

### *BCRP/ABCG2* expression in JAR cells measured by V-BCRPi immunofluorescence

After infecting JAR cells with each recombinant V-BCRPi retrovirus for 48 hours, the cells were removed from the incubator. Groups of uninfected cells incubated with primary antibody or PBS were used as controls in this set of experiments. The cells were washed three times with warm 1× PBS for 10 minutes; then, 4% cold paraformaldehyde was added, and the cells were incubated at room temperature for 20 to 30 minutes. The cells were then washed three times with 1× PBS for 10 minutes each. Next, 0.2% Triton X-100 was added for 10 minutes to permeabilize the cells, and the cells were washed three times with 1× PBS for 10 minutes each. The cells were incubated with serum for blocking at room temperature for 30 minutes. The cells were then incubated with primary antibody (diluted in 1% BSA) in a humidified box for 4 nights and washed three times with 1× PBS for 10 minutes each. The secondary antibody, which was labeled with fluorescein (diluted in 1% BSA), was added to the cells for 30 minutes in the absence of light, and the cells were then washed three times with 1× PBS for 10 minutes each. DAPI staining was performed and observed under a fluorescence microscope after the cells were mounted on slides.

### FCM analysis of the influence of V-BCRPi on *BCRP/ABCG2* function

JAR cells were plated in a six-well plate and infected with each recombinant V-BCRPi retrovirus for 48 hours with 70% confluent. The medium was exchanged, mitoxantrone was added at a final concentration of 3 µM, and the cells were cultured in an incubator for an additional 2 hours. The cells were washed with PBS, the media were exchanged, and the cells were cultured for an additional hour. The cells were then trypsinized and resuspended in PBS. The amount of mitoxantrone fluorescence that was retained in 5,000 cells was then determined using flow cytometry. An excitation wavelength of 488 nm and an emission wavelength of 675 nm were used.

### The JAR transplant subcutaneous sarcoma model

The cell-grade malignancy and tumor-formation ratios of the JAR cells were high, and endogenous *BCRP/ABCG2* was highly expressed. The JAR cells were cultured until they reached 80–90% confluency. The cells were detached using 0.25% trypsin and were collected in a single-cell suspension in serum-free RPMI-1640 medium; 0.2 ml of the suspension (4–8×10^6^ JAR cells) was injected under the dorsal skin of a hairless mouse to form a subcutaneous tumor. The mice were observed every day for tumor growth, swelling, and diabrosis. The following experiment was conducted after the tumors reached 15–75 mm^3^ in size.

### *In vivo* analysis of tumor-bearing hairless mice

The hairless mice were divided into four groups, each containing 15 mice: 1: a blank control group that was injected with 200 µl of sterile PBS; 2: a group injected with 200 µl of sterile PBS into the tumor body and 200 µl of 10× IC_50_ of the drug into the abdominal cavity; 3: a group injected with 200 µl of V-BCRP3i viral suspension into the tumor body; and 4: a group injected with 200 µl of V-BCRP3i viral suspension into the tumor body and 200 µl of 10× IC_50_ of the drug into the abdominal cavity. The intraperitoneal injections took place 48 hours after the injection of the viral suspension into the tumor body. The mice were observed for 14 days and then euthanized by cervical dislocation on the 15^th^ day and reserved as specimens. Both tumor volume (V) and tumor weight (g) were both measured to calculate the tumor inhibition ratio: tumor inhibition ratio = (1−G/g)×100%, where G is the tumor weight of the treatment group and g is the tumor weight of the control group. The tumor volume (V) was calculated using the following equation: Tumor volume (V) = length×0.5 width. After 10 blocks of each group were quickly soaked in liquid nitrogen, the samples were stored at −70°C in a cryogenic refrigerator until they were used to measure variations in the mRNA and protein levels. The 5 remaining blocks from each group were fixed with 10% formalin.

### Immunohistochemistry

After fixation with 10% formalin and paraffin-embedding, clinical breast cancer and placental tissue slices were studied using immunohistochemistry. After the paraffin was removed and the slides were hydrated, endogenous peroxidase was blocked. The samples were microwaved for antigen retrieval. The mouse-anti-human BCRP/ABCG2 antibody (BXP-21) was diluted 1∶50 and incubated with the samples overnight at 4°C. The primary antibody was replaced with PBS to serve as a blank control. A rabbit-anti-mouse horseradish peroxidase-conjugated secondary IgG antibody was added to the samples and incubated for one hour at room temperature. The slices were then washed with PBS and developed with diaminobenzidine (DAB). The slices were then re-stained with hematoxylin and stripped of color with ethanol. Dimethylbenzene was used to clean and seal the samples, which were then observed under a microscope. The section staining was repeated three times.

### In situ apoptosis analysis using the TUNEL assay

The paraffin-embedded tissue slices were washed with dimethylbenzene, dehydrated with ethanol, fixed with formalin, treated with proteinase K, fixed with formaldehyde solution, and washed with PBS. In total, 50 µl of TdT was added to the samples for 5 minutes at room temperature, and the samples were then incubated at 37°C in a humid environment for 90 minutes. The samples were washed three times with PBS, bathed in H_2_O_2_ for 3 to 5 minutes, and then washed again with PBS. The samples were then incubated with streptavidin-HRP solution for 3 to 5 minutes at room temperature and then washed with PBS three times. Next, 100 µl of DAB working solution was applied until a light brown color appeared; the samples were then rinsed with deionized water and cleaned with ethanol for 3 minutes. The samples were fixed with dimethylbenzene, sealed with resin, and observed under a microscope; photographs of the samples were taken. Six random views were selected for each slice. The number of brown positive cells for every 50 cells within each view was counted, and the apoptosis index (AI), which reflects the degree of apoptosis, was calculated using the following equation: AI (TUNEL) = (total positive cells/300)×100%.

### Statistical analysis

The data are represented as the mean±standard deviation (SD) and were analyzed using one-way ANOVA analysis. *P*<0.05 was considered statistically significant.

## Results

### Sequencing results of the RNA interference oligonucleotide construct targeting the *BCRP/ABCG2* ATP-binding domain

Three groups of pLenti6/BLOCK-iT constructs bearing unique restriction sites and DNA sequences complementary to the ABC domain of the *BCRP/ABCG2* gene were designed using the BLOCK-iT RNAi Designer (Invitrogen). These fragments began from the 207^th^, 745^th^ and 939^th^ initiation sites of the 6^th^ exon and were named BCRP1i, BCRP2i, and BCRP3i, respectively. The sequences of each pair of DNA oligonucleotides are as follows:

Beginning at the 207^th^ bp of the BCRP1i ATP-binding domain (20 bp):

5′CACCGCAGGATAAGCCACTCATAGACGAATCTATGAGTGGCTTATCCTGC3′
5′AAAAGCAGATGCCTTCTTCGTTATGTTCGTCTATGAGTGGCTTATCCTGC3′

Beginning at the 745^th^ bp of the BCRP2i ATP-binding domain (20 bp):

5′CACCGCTTCAGTACTTCAGCATTCCCGAACATAACGAAGAAGGCATCTGC3′
5′AAAAGCAGATGCCTTCTTCGTTATGTTCGCATAACGAAGAAGGCATCTGC3′

Beginning at the 939^th^ bp of the BCRP3i ATP-binding domain (20 bp):

5′CACCGCTTCAGTACTTCAGCATTCCCGAAGGAATGCTGAAGTACTGAAGC3′
5′AAAAGCTTCAGTACTTCAGCATTCCTTCGGGAATGCTGAAGTACTGAAGC3′

### Preparation of the pENTR/U6-BCRPi entry vectors and recombinant pLenti6/BCRPi retroviruses

After plasmid extraction, sequencing and purification, three groups of positive pENTR/BCRPi recombinant plasmids were obtained and named as follows: pENTR/BCRP1i, pENTR/BCRP2i, and pENTR/BCRP3i. Additionally, one clone of each base mutation was selected to serve as a control group, and these constructs were named pENTR/BCRP1i-C, pENTR/BCRP2i-C, and pENTR/BCRP3i-C. After the LR enzymatic reaction, six groups of positive pLenti6/BCRPi recombinants were obtained: pLenti6/BCRP1i, pLenti6/BCRP1i-c, pLenti6/BCRP2i, pLenti6/BCRP2i-c, pLenti6/BCRP3i, and pLenti6/BCRP3i-c. After transfection of the 293FT cells, retrovirus preparation, and titering, each group of retroviruses achieved a 10^6^ titer, and these samples were used for the subsequent *in vitro* and *in vivo* infection experiments.

### Drug sensitivity of V-BCRPi-enriched cells measured by cell survival and the JAR cell inhibition ratio

**Figure 1:**
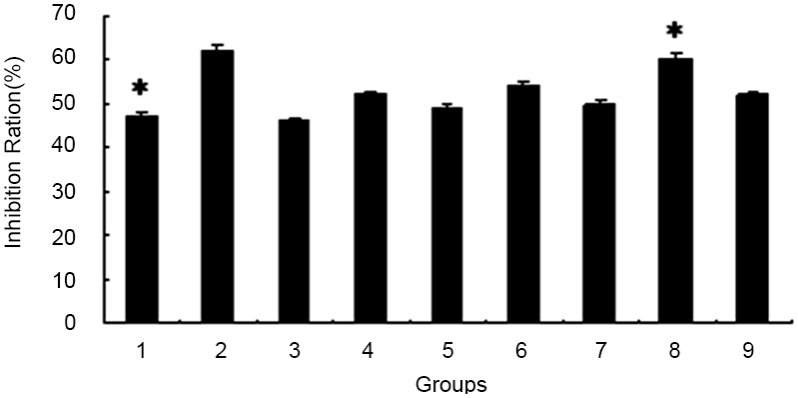
Inhibition ratio of 5-FU against JAR cells infected with V-BCRPi using cell survival analysis. Experimental groups 1 to 9, which were submitted to the following treatments, are shown: 1: 5-FU, 2: Ko143+5-FU, 3: pLenti6/vector+5-FU, 4: V-BCRP1i+5-FU, 5: V-BCRP1ic+5-FU, 6: V-BCRP2i+5-FU, 7: V-BCRP2ic+5-FU, 8: V-BCRP3i+5-FU, and 9: V-BCRP3ic+5-FU. The inhibition ratio of the V-BCRP3i treatment group was significantly higher than those of the other virus-free treatment groups. Each inhibition ratio represents the mean value of three independent experiments. *P<0.01.

The inhibition ratio of each V-BCRPi-treated cell group greatly improved. However, the ratio of the V-BCRP3i treatment group was significantly higher than those of the virus-free treatment groups (*P*<0.01). Moreover, there was little difference between the specific inhibitor Ko143 group. In addition, it was discovered that the cell inhibition ratios of the V-BCRPi-c and V-BCRPi groups were reduced (Fig. 1).

### Knockdown of *BCRP/ABCG2* expression by V-BCRPi in JAR cells, measured by fluorescent quantitative RT-PCR

The expression of *BCRP/ABCG2* in each V-BCRPi treatment group was lower than that of the blank cell group; however, the levels of *BCRP/ABCG2* expression in the no expression vector and blank cell groups were similar. Among the V-BCRPi groups, the expression of *BCRP/ABCG2* in the V-BCRPi-c group was the highest. The inhibition ratio of the expression of *BCRP/ABCG2* in the V-BCRP3i treatment group was the lowest but was still significantly different from the blank cell group (*P*<0.01).

### Knockdown of BCRP/ABCG2 expression by V-BCRPi in JAR cells, as determined by Western blot analysis

**Figure 2:**
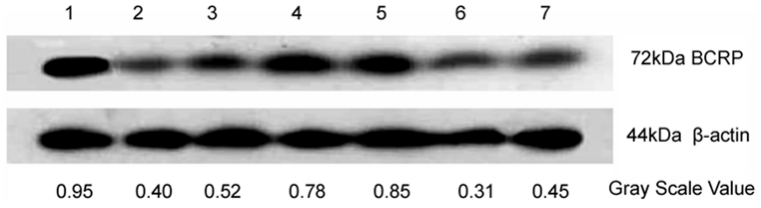
Knockdown of BCRP/ABCG2 expression by V-BCRPi in JAR cells, shown by Western blot analysis. The results for experimental groups 1 to 7 are shown: 1: mock cells or cells infected with 2: V-BCRP1i, 3: V-BCRP1i-c, 4: V-BCRP2i, 5: V-BCRP2i-c, 6: V-BCRP3i, or 7: V-BCRP3i-c. The BCRP/ABCG2 protein expression of the V-BCRP3i treatment group was the lowest (group 6).

After 48 hours of infection by V-BCRPi, total cellular protein was extracted and analyzed. The BCRP/ABCG2 protein expression in each V-BCRPi-treated group was lower than that of the blank cell group. Of all of the V-BCRPi-treated groups, the expression of BCRP/ABCG2 was highest in the V-BCRPi-c-treated group and lowest in the V-BCRP3i treatment group (Fig. 2).

### Knockdown of BCRP/ABCG2 by V-BCRPi in JAR cells, as measured by immunofluorescence

**Figure 3:**
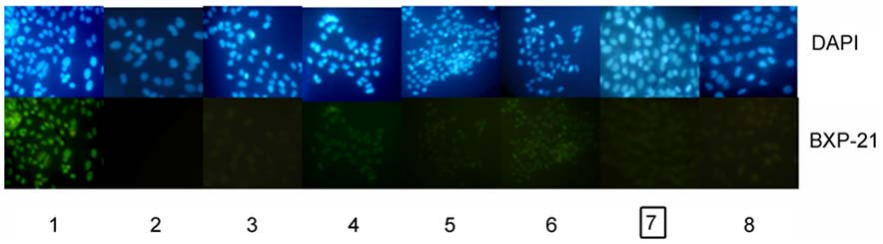
Knockdown of BCRP/ABCG2 expression by V-BCRPi in JAR cells using immunofluorescence analysis (×100). The results for experimental groups 1 to 8 are shown: 1: mock cells with MoAb or 2: PBS or cells infected with 3: V-BCRP1i, 4: V-BCRP1i-c, 5: V-BCRP2i, 6: V-BCRP2i-c, 7: V-BCRP3i, or 8: V-BCRP3i-c. The fluorescence intensity of cells subjected to V-BCRP3i treatment was the lowest.

After 48 hours of infection by V-BCRPi, immunofluorescence was used to analyze the cell morphology. Using DAPI staining for comparison, the fluorescence intensity of BCRP/ABCG2 in each V-BCRPi treatment group was reduced; the fluorescence intensity of uninfected cells incubated with the primary antibody was the strongest, and the fluorescence intensity of uninfected cells with PBS instead of the primary antibody was weakest. The fluorescence intensity of the V-BCRPc treatment group was higher than those of all the other V-BCRPi groups, and the fluorescence intensity of the V-BCRP3i treatment group was the lowest (Fig. 3).

### Influence of V-BCRPi on the BCRP/ABCG2 drug pump, as measured by FCM

**Figure 4:**
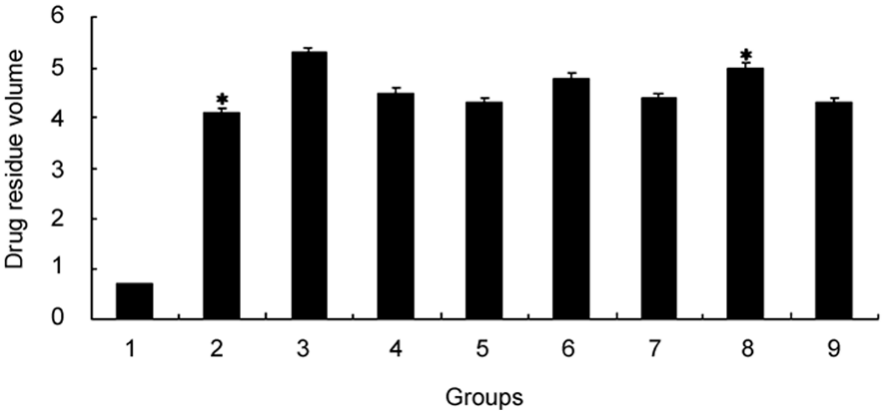
Residual drug volumes after infection of JAR cells with V-BCRPi according to flow cytometry analysis. The results of experimental groups 1 to 9 are shown: 1: mock cells, 2: cells with Mit, 3: cells with Mit and Ko143, or cells with Mit and infected with 4: V-BCRP1i, 5: V-BCRP2i, 6: V-BCRP3i, 7: V-BCRP1i-c, 8: V-BCRP2i-c, or 9: V-BCRP3i-c. Each residual drug volume represents the mean value of three independent experiments. *P<0.01.

The results indicated that, after infection with the recombinant V-BCRpi retroviruses, the amount of mitoxantrone that was retained by the cells was increased but was similar to that of the blank control group. The amount of drug retained after infection with V-BCRP3i was greatly improved, with a mean fluorescence intensity of 4.9 (*P*<0.01). Infection with the recombinant retroviruses with single base variations resulted in lower retention than that observed in the V-BCRPi-infected cells, but the retention was higher than that of the blank cell control group. The mean value of the fluorescence intensity for the Ko143 group (positive control) was 5.3 (Fig. 4). It was therefore concluded that the increased amount of drug within the V-BCRP3i-infected JAR cells would increase the effect of the drug on the cell.

### Results of tumor growth after V-BCRPi and 5-FU injection

**Figure 5:**
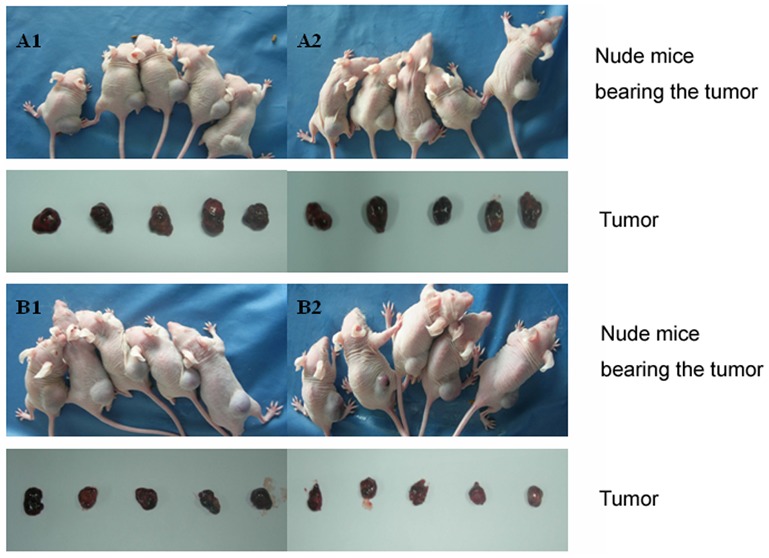
Tumor bodies of hairless mice after injection of JAR cancer cells infected with V-BCRPi and treatment with 5-FU. A1: Tumor body injected with PBS alone; A2: Tumor body injected with PBS and 5-FU; B1: Tumor body injected with V-BCRPi alone; and B2: Tumor body injected with V-BCRPi and 5-FU. After injecting 5-FU, the tumors in the hairless mice injected with V-BCRPi were smaller than those in the un-injected hairless mice. The anti-tumor rate was approximately a factor of 10 (*P*<0.01). It was concluded that V-BCRPi increases the inhibition effects of 5-FU on tumor growth.

On the 10^th^ and 12^th^ days of inoculation, we observed a grain-sized node at the injection site of the JAR cells. A transplantation tumor was formed in mice from each group, with a 100% success rate. We observed the tumors on the 14^th^ day after injecting V-BCRPi into the tumor and 10× IC_50_ of 5-FU into the peritoneal cavity. The transplantation tumors grew quickly in elliptical shapes during the early stages and transformed into smooth and irregular forms with nodositas during the later stages. The covering skin was purplish-black. When the diameter of the tumor was greater than 1 cm, the ulceration began to feel soft and cystic. When the skin was removed from the transplantation tumor, the surface of the solid tumor tissue was red-brown. The hairless mice injected with 5-FU alone did not eat and appeared dispirited at an early stage. At the later stages, the mental state and appetites of the mice improved. After injecting 5-FU, the tumors in the hairless mice previously injected with V-BCRPi were smaller than those of hairless mice that were not injected with the virus. The anti-tumor rate was approximately 10 times as slow (*P*<0.01; Fig. 5 and Table 1). It was therefore concluded that V-BCRPi increases the 5-FU inhibition effects on tumor growth.

**Table 1:** Changes in tumor weight and volume and the anti-tumor rate in transplantation tumors in nude mice after virus infection and drug treatment (x±s, n = 15).

Group	Tumor weight (g)	Tumor volume (cm3)	Anti-tumor rate (%)
PBS	2.46±0.56	3.71±0.73	-
*PBS+5-FU	2.37±0.78	3.62±0.56	3.6
V-BCRPi	2.31±0.49	3.65±0.53	-
*V-BCRPi+5-FU	1.75±0.65	2.89±0.57	24.2

*: PBS+5-FU vs. V-BCRPi+5-FU, p<0.01.

### Knockdown of *BCRP/ABCG2* and drug sensitivity by V-BCRPi in hairless mice bearing tumors, as shown using immunohistochemical analysis

**Figure 6:**
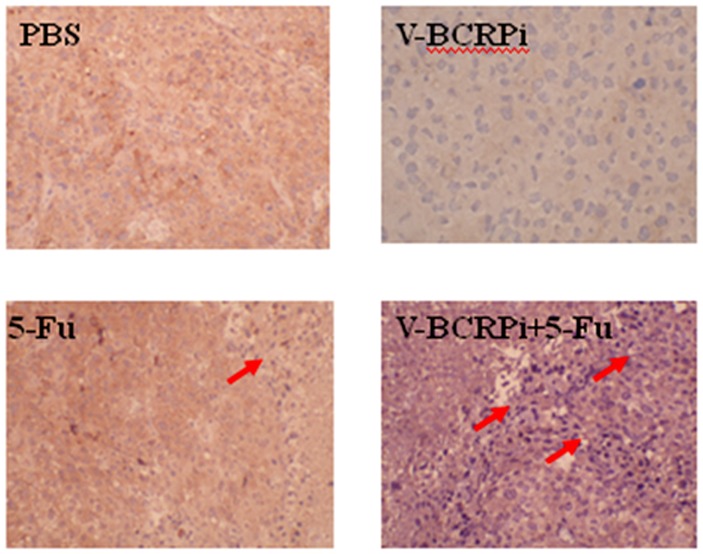
Immunohistochemical staining of tumor bodies in hairless mice bearing JAR cancer cells after injection with V-BCRPi and 5-FU (×100). The results indicate that V-BCRPi inhibits *BCRP/ABCG2* expression and improves drug sensitivity to 5-FU in tumors. The red arrows indicate the dead cells.

The expression of *BCRP/ABCG2* in tumors injected with V-BCRPi was lower than that in tumors not injected with V-BCRPi (including tumors injected with PBS and 5-FU alone). The membranes of the *BCRP/ABCG2-*positive histiocytes were brown, and the nuclei were blue, with larger karyoplasm and altered morphologies in different cells. Of the groups injected with 5-FU, more dying cells were evident in the groups injected with V-BCRPi than in the uninjected group. Specifically, cellular morphology was lost, the karyoplasm was large, and necrosis was increased in the virus-infected cells (Fig. 6). These results indicate that V-BCRPi inhibits *BCRP/ABCG2* expression in tumor cells and improves their sensitivity to 5-FU.

### Apoptosis of V-BCRPi-treated tumor cells in hairless mice, as demonstrated by in situ hybridization analysis

**Figure 7:**
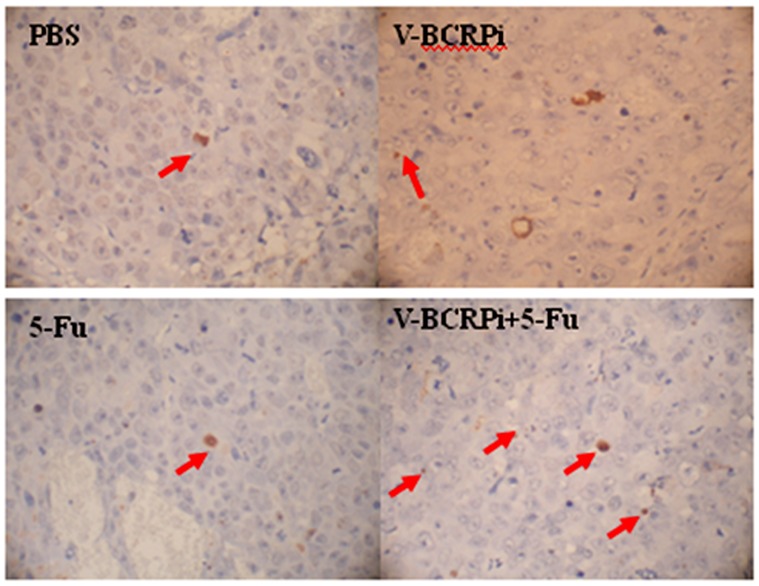
TUNEL staining of the tumor bodies of hairless mice bearing JAR cancer cells injected with V-BCRPi and 5-FU (×200). The number of apoptotic cells in tumors injected with V-BCRPi and 5-FU was greater than that of the tumors injected with 5-FU alone. The red arrows indicate the apoptotic cells.

Each group of tumor tissue slices was stained with TUNEL and re-stained with hematoxylin. After staining, the cells with brown nuclei of different sizes and irregular forms were considered TUNEL-positive, apoptotic cells. The single blue cells were non-apoptotic chorion cancer cells. The results indicated that the amount of apoptotic tumor cells in mice injected with V-BCRPi and 5-FU was greater than that in mice injected with 5-FU alone (Fig. 7). According to the calculation results, the number of apoptotic tumor cells following injection with V-BCRPi and 5-FU was larger than the number of apoptotic tumor cells following injection with 5-FU alone (Table 2).

**Table 2:** Changes in the apoptosis rate of transplantation tumors in nude mice injected with virus and 5-FU (x±s, n = 5).

Groups	Apoptosis rate (%)
PBS	5.71±0.43
*PBS+5-FU	17.62±0.50
V-BCRPi	7.65±0.35
*V-BCRPi+5-FU	22.78±0.47

*: PBS+5-FU vs. V-BCRPi+5-FU, p<0.01.

### Knockdown of *BCRP/ABCG2* in hairless mice bearing V-BCRPi-treated tumors, as shown by Western blot analysis

**Figure 8:**
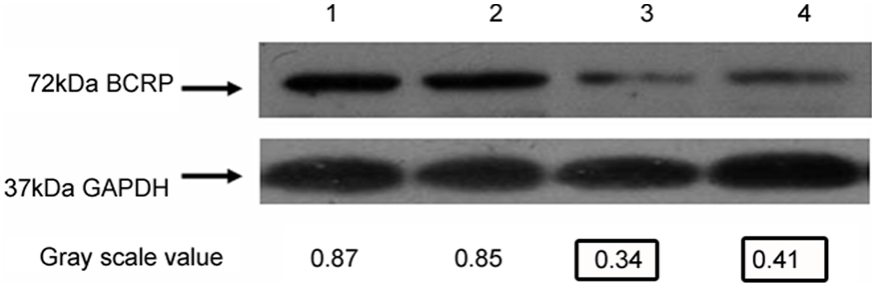
Knockdown of BCRP/ABCG2 expression by V-BCRPi in tumor cell bodies, as shown by Western blot analysis. 1: Tumor body injected with PBS alone; 2: Tumor body injected with PBS and 5-FU; 3: Tumor body injected with V-BCRPi alone; and 4: Tumor body injected with V-BCRPi and 5-FU. It was concluded from the experimental results that the expression of *BCRP/ABCG2* in tumors injected with V-BCRPi (with 5-FU treatment) was lower than that of the un-injected tumors (with PBS and 5-FU). There was no difference among the groups injected with the various V-BCRPi retroviruses and 5-FU.

Based on the results, the following conclusions were made. The expression of *BCRP/ABCG2* in V-BCRPi-injected tumors (including those injected with 5-FU) was lower than that of uninjected tumors (including those injected with PBS and 5-FU). There were no differences between the groups injected with V-BCRPi and 5-FU. The BCRP/ABCG2 protein expression level was lower in the non-injected group (Fig. 8).

### Knockdown of *BCRP/ABCG2* in hairless mice bearing V-BCRPi-treated tumors, as shown by fluorescent quantitative RT-PCR analysis

The results showed that the *BCRP/ABCG2* mRNA level in the V-BCRPi-injected group was lower than that in the group injected with 5-FU alone (*P*<0.01). The mRNA expression in the group injected with V-BCRPi alone was not different from that of the group injected with 5-FU alone (Table 3). It was therefore concluded that V-BCRPi could more efficiently downregulate *BCRP/ABCG2* mRNA in tumor histiocytes.

**Table 3:** Relative BCRP/ABCG2 transcript levels of the transplantation tumors in nude mice injected with virus and drugs (x±s, n = 5).

Group	Relative transcript level
PBS	1
*PBS+5-FU	0.70±0.05
V-BCRPi	0.20±0.03
*V-BCRPi+5-FU	0.08±0.05

*: PBS+5-FU vs. V-BCRPi+5-FU, p<0.01.

## Discussion

Since the first report of RNA interference (RNAi) in 1998, its feasibility and innovation have been continuously improved. RNAi has shown the advantages of high efficiency, specificity, simple operation and a short experimental period [14], [15], and it has become a powerful tool for gene function and gene therapy research [16]. *In vitro* chemical synthesis has been used from the early stages of the development of the technique. The techniques of in vitro transcription, siRNA expression cassettes, and *in vivo* transcription for PCR preparation were middle stage developments [17]. More recently, stable expression DNA carriers have proven advantageous for the *in vivo* transcription of siRNA. Targeted gene inverted repeat sequences have been placed downstream of the promoters of retroviral expression vectors to create recombinant viruses with short hairpin loops (shRNA), and these constructs have resulted in the best inhibition effects to date [18], [19]. This type of viral vector has long-lasting RNAi effects that can infect a wide range of cells, thus increasing the potential applications of this technology.

Some researchers have applied the *in vitro* chemical synthesis siRNA method to explore the reversal of P-gp-mediated multidrug resistance, which has provided a basis for the operability of this technique [20]. The pLenti6/BLOCK-iT lentiviral RNAi expression system is an RNAi system expressed by a 3^rd^ generation, replication-defective retrovirus that was developed by Invitrogen. This system has been widely applied in scientific research on gene functions and clinical gene biotherapy due to its efficient expression and infection and high biosafety and operability. One goal of researchers is to counter the drug resistance of tumor cells and improve drug sensitivity by modifying the BCRP/ABCG2-mediated, drug-resistant phenotype and the drug-resistant phenotypes of ABC membrane transporter families. Therefore, we first used the pLenti6/BLOCK-iT lentiviral RNAi expression system to construct vectors targeting conserved regions of the ATP-binding domain of *BCRP/ABCG2* and then conducted *in vitr*o and *in vivo* RNA interference studies [21]. In this study, three DNA constructs that were complementary to the ABC domain of the *BCRP/ABCG2* gene, beginning at the 207^th^, 745^th^ and 939^th^ bps of the 6^th^ exon of the *BCRP/ABCG2* gene [22]–[24], were successfully designed. Subsequently, these fragments were used to construct pENTR/BCRPi recombinant plasmids through annealing, recombination, transformation, sequencing and comparison using BLAST. The pLenti6/BLOCK-iT Lentiviral RNAi expression vectors were then constructed. After 293T virus packaging, highly infective retroviruses were acquired with titers of 10^6^. In the subsequent *in vivo* and *in vitro* infection studies, we expected to screen for biotherapy reversal agents that could reverse drug resistance by efficiently targeting the ATP-binding domain. This method will lay a solid foundation for future studies and applications.

BCRP/ABCG2, P-gp and multidrug resistance-associated proteins (MRPs) all have ABC domains, which use ATP hydrolysis to “pump” drugs stored inside cancer cells outside of the cell membrane, thus facilitating drug resistance [25]–[32]. Determining how to reverse the multidrug resistance mediated by drug-resistance proteins is a problem that must be solved. RNAi using DNA constructs encoding siRNAs that are complementary to the ABC domain of *BCRP/ABCG2* has shown that gene silencing of multidrug-resistant proteins is possible [33].

In this study, multiple RNA hairpins were designed to target the ABC domain of *BCRP/ABCG2*, and stable shRNA-expressing recombinant retroviruses with high infection rates were constructed [34]–[36]. Using this RNA interference system with *in vitro* cell studies, the siRNA recombinant retroviruses were screened for efficient knockdown of *BCRP/ABCG2* mRNA and protein expression levels and alterations in the drug susceptibility phenotype using immunofluorescence and drug pump functional tests. It was concluded that all three groups of recombinant retroviruses that targeted the ABC domain (including recombinant retroviruses with single base mutations) efficiently knocked down *BCRP/ABCG2*. The 3^rd^ group, V-BCRP3i, exhibited the best knockdown effects. Additionally, the results indicated that a single base change in the target sequence resulted in an siRNA interference efficiency that was lower than that of the corresponding non-mutated experimental group. This finding further demonstrates the efficiency and sequence specificity of siRNA interference. Therefor, this study screened for cells with better *in vitro* interference effects caused by the V-BCRPi retroviruses targeting the *BCRP/ABCG2-*mediated drug-resistant phenotypes and other ABC transporter protein-mediated drug-resistant phenotypes. This study also demonstrated how to conduct *in vivo* studies and overall assessments in animals, as well as how to design efficient, specific siRNAs targeting ABC domains to reverse drug resistance and improve cell sensitivity to drugs.

Heterotransplantation in hairless mice is a favorable *in vivo* animal model to study cancer treatment because experimental results using transplantation tumors in these mice feasibly predict clinical effects. When exploring drug resistance mechanisms and the reversal of drug resistance, the hairless mouse heterotransplantation tumor model has important implications for further studies of the prognosis and survival rates for patients with drug-resistant tumors.

The growth of transplanted tumors in hairless mice is affected by the biological characteristics of the tumors and the body features of the hairless mice. This study inoculated 10^6^ highly malignant JAR cells expressing high levels of endogenous *BCRP/ABCG2* into hairless mice. The results showed strong cell reproductive capacity, early formation of the transplantation tumors and a tumor formation rate of 100%, which meant that the transplantation tumor model was successfully established.

Overcoming multidrug resistance in tumors would improve chemotherapy and comprehensive treatment effects, survival rates and quality of life. In recent years, RNAi interference techniques have provided favorable strategies for improving the multidrug resistance of tumors, especially when using siRNA retroviral vectors with high infectability, specificity and stability. In this study, the pLenti6/BLOCK-iT Lentiviral RNAi expression system was used to construct recombinant retroviruses (V-BCRPi) that were then used *in vitro* to determine which retrovirus conferred the best knockdown effects and improved the *BCRP/ABCG2*-mediated drug-resistant phenotype. An animal *in vivo* study was then conducted using transplanted tumors in hairless mice. V-BCRPi injection into tumor histiocytes followed by mRNA and protein level analysis showed that *BCRP/ABCG2* expression was inhibited. This study also revealed the improved function of the drugs, the induction of cell death and the reversal of drug resistance in the transplantation tumors, which were demonstrated by decreased transplantation tumor growth and drug sensitivity.

The study results provide a favorable experimental animal model for the reversal of ABC membrane transporter protein family-mediated drug resistance and a scientific basis for targeting the ABC domain by V-BCRPi with high efficiency and specificity.

Although *in vitr*o and *in vivo* experiments have demonstrated the favorable prospects of applying RNAi interference in gene treatment, functional gene research and drug sensitization, the safe administration of the siRNAs to humans and the expression of these constructs in specific organs and tissues in clinical applications still need to be perfected. In addition, the expression of *BCRP/ABCG2* in normal tissues, such as placenta, brain, prostate, small intestine, testicles, ovary, colon and liver, is high. *BCRP/ABCG2* plays a fundamental role in maintaining normal physiological functions and preventing damage from external toxins. Determining how to improve the drug sensitivity of *BCRP/ABCG2* in selected RNAi-targeted tumor cells still remains to be elucidated.
