# Correction: Modulating Drug Resistance by Targeting *BCRP/ABCG2* Using Retrovirus-Mediated RNA Interference

**DOI:** 10.1371/journal.pone.0111279

**Published:** 2014-10-10

**Authors:** The PLOS ONE Staff

The seventh author’s name is spelled incorrectly. The correct spelling is: Yi Jing.
